# Epidemiologic Characteristics of Chronic Hepatitis B and Coinfections with Hepatitis C Virus or Human Immunodeficiency Virus in South Korea: A Nationwide Claims-Based Study Using the Korean Health Insurance Review and Assessment Service Database

**DOI:** 10.3390/pathogens14070715

**Published:** 2025-07-19

**Authors:** Hyunwoo Oh, Won Sohn, Na Ryung Choi, Hyo Young Lee, Yeonjae Kim, Seung Woo Nam, Jae Yoon Jeong

**Affiliations:** 1 Department of Internal Medicine, Kangbuk Samsung Hospital, Sungkyunkwan University School of Medicine, Seoul 03181, Republic of Korea; asklepios1258@gmail.com; 2 Department of Internal Medicine, Ewha Womans University Mokdong Hospital, Ewha Womans University College of Medicine, Seoul 07985, Republic of Korea; lemonsmilk87@hanmail.net; 3 Department of Internal Medicine, Hanyang University Hospital, Hanyang University College of Medicine, Seoul 04763, Republic of Korea; catchhyong@gmail.com; 4 Department of Internal Medicine, National Medical Center, Seoul 04564, Republic of Korea; arckyj@nmc.or.kr (Y.K.); kn37503@hotmail.com (S.W.N.)

**Keywords:** chronic hepatitis B, hepatitis C virus, human immunodeficiency virus, prevalence, comorbidity, health insurance review and assessment service

## Abstract

Coinfections with hepatitis C virus (HCV) or human immunodeficiency virus (HIV) among individuals with chronic hepatitis B (CHB) are associated with worse clinical outcomes but remain understudied due to their low prevalence and the sensitivity of associated data. This nationwide, cross-sectional study utilized claims data from the Korean Health Insurance Review and Assessment Service (2014–2021) to investigate the prevalence, comorbidities, treatment patterns, and liver-related complications among patients with HBV monoinfection, HBV/HIV, HBV/HCV, or triple coinfection. Among over 4.5 million patients with chronic hepatitis B, the prevalence of HIV and HCV coinfection ranged from 0.05 to 0.07% and 0.77 to 1.00%, respectively. Patients with HBV/HCV coinfection were older and had significantly higher rates of hypertension, diabetes, dyslipidemia, and major adverse liver outcomes, including hepatocellular carcinoma and liver transplantation, compared to other groups. HBV/HIV coinfection was more common in younger males and was associated with higher dyslipidemia. The use of HBV antivirals increased over time across all groups. These findings highlight the distinct clinical characteristics and unmet needs of coinfected populations, underscoring the importance of tailored screening and management strategies in HBV-endemic settings.

## 1. Introduction

The World Health Organization (WHO) estimates that 254 million people were living with chronic hepatitis B (CHB) infection in 2022, resulting in 1.1 million deaths, mainly from cirrhosis and liver cancer [1]. Hepatitis B virus (HBV), hepatitis C virus (HCV), and human immunodeficiency virus (HIV) share common modes of transmission, including blood exposure, sexual contact, and vertical transmission from mother to child [2]. In patients with CHB, the prevalence of coinfection with HIV or HCV has been reported to vary from 1% to 15% [1,3]. This variation across studies is influenced by factors such as population demographics [4] and regional epidemiology [5,6,7]. The 2022–2030 Global Health Sector Strategies, endorsed by the WHO, aim to end the epidemics of HIV, viral hepatitis (B and C), and sexually transmitted infections by 2030 through people-centered, rights-based, and integrated approaches [8]. With the development and global implementation of antiretroviral therapy (ART) for HIV, direct-acting antivirals (DAAs) for HCV, and nucleos(t)ide analogues (NAs) for HBV, the prevalence of viral coinfections has declined in some populations in recent years [9,10]. However, unlike HBV, neither HCV nor HIV has a preventive vaccine, resulting in a disproportionately higher burden among individuals engaging in unsafe injecting practices [2,11]. Moreover, the prevalence of hepatitis B and C infections tends to increase in parallel with that of HIV, reflecting their common modes of transmission [12]. Coinfection with HIV or HCV is associated with the accelerated progression of HBV infection to liver fibrosis and hepatocellular carcinoma (HCC), leading to increased morbidity and mortality. Patients with coinfection also exhibit reduced treatment response, a higher risk of drug-induced hepatotoxicity, and potential cross-resistance to antiviral agents due to overlapping therapeutic regimens [6,13,14].

However, there is a paucity of research on the epidemiology, treatment patterns, and liver-related complications associated with CHB coinfection with either HCV or HIV, particularly in South Korea. Due to the relatively low prevalence of these coinfections and the sensitive nature of the associated conditions, most existing studies have been limited by small sample sizes [15,16], and large-scale analyses addressing comorbidities and healthcare utilization among coinfected patients remain scarce. Therefore, this study aimed to investigate the prevalence, comorbidities, treatment patterns, and liver-related complications among patients with CHB and HCV or HIV coinfection, utilizing nationwide claims data from the Health Insurance Review and Assessment Service (HIRA) in South Korea. In addition, a comparative analysis of these parameters was conducted over an eight-year period against a reference group of patients with HBV monoinfection.

## 2. Materials and Methods

### 2.1. Study Design and Data Source

This study was a retrospective, population-based study performed using cross-sectional data from HIRA in South Korea. The HIRA database contains health insurance claims data that are also called national health insurance (NHI) data as they are generated in the process of reimbursing claims for healthcare services under the NHI system in Korea [17]. It includes healthcare data for nearly the entire Korean population, such as demographics, in-hospital treatment, diagnoses, prescription, and provider characteristics. The NHI system has covered almost 97% of the total population, which numbered approximately 50 million as of 2014 in Korea [18].

### 2.2. Study Population and Clinical Variables

This study aimed to investigate differences in the epidemiology of Korean patients with HBV monoinfection, HBV/HIV coinfection, HBV/HCV coinfection, and HBV/HIV/HCV coinfection. Disease diagnosis was defined based on the International Statistical Classification of Diseases and Related Health Problems, Tenth Revision (ICD-10) diagnosis codes with the data on procedures and prescriptions in the HIRA database. During the study period, the number of individuals aged ≥20 years enrolled in the National Health Insurance program was 41,166,634 in 2014; 41,701,269 in 2015; 42,200,854 in 2016; 42,653,484 in 2017; 43,080,834 in 2018; 43,699,187 in 2019; 44,048,160 in 2020; and 44,390,488 in 2021.

Age and sex were included in the analysis as demographic characteristics. Age was categorized into five groups: “20–39 years”, “40–49 years”, “50–59 years”, “60–69 years”, and “70 years and older”. Sex was categorized into “Male” and “Female”. The study population included patients with chronic hepatitis B (ICD-10: B18.0, B18.1, Z22.5) aged over 20 years between 1 January 2014 and 31 December 2021. We excluded patients with hepatitis D virus infection (ICD-10: B18.0) and other chronic viral hepatitis (ICD-10: B18.8, B18.9). We classified patients with chronic hepatitis B into four groups: (1) HBV monoinfection, (2) HBV/HIV coinfection, (3) HBV/HCV coinfection, and (4) HBV/HIV/HCV coinfection. HCV infection (ICD-10: B18.2) and HIV infection (ICD-10: B20-24 and Z21) were also defined based on ICD-10 codes.

This study compared the differences in clinical variables between four groups by years, namely 2014–2021. Also, we investigated the trends of clinical outcomes among four groups from 2014 to 2021. The clinical variables were age, sex, presence of liver-related outcomes such as HCC, liver transplantation, esophageal or gastric varix, variceal hemorrhage, spontaneous bacterial peritonitis (SBP), hepatorenal syndrome, and presence of comorbidities such as hypertension (HTN), diabetes mellitus (DM), and dyslipidemia (DLD) with the Charlson Comorbidity Index (CCI) score; comorbidities were defined using ICD-10 codes and summarized using the CCI [19]. We also compared the use of antiviral agents for HBV infection, HCV infection, and HIV infection. The definition of clinical variables and antiviral agents was as follows. Clinical outcomes were defined as ascites (ICD-10: R18 or use of diuretics [spironolactone, amiloride, furosemide, and torasemide], variceal bleeding (ICD-10: I85.0, I85.0, I85.0, I85.0, and I85.0 with endoscopic variceal ligation or sclerotherapy), hepatic encephalopathy (ICD-10: G93.4, G31.2, K72.01, K72.11, and K72.91), spontaneous bacterial peritonitis (ICD-10: K65.0, and K65.9), and hepatorenal syndrome (ICD-10: K76.7). The definition of esophageal or gastric varix was based on the diagnosis (ICD-10: I85.0, I85.2-I85.4, I85.9) or the use of non-selective beta-blockers (propranolol, carvedilol, and nadolol). Antiviral treatment was defined as the prescriptions of each antiviral agent for HBV (lamivudine, adefovir, telbivudine, entecavir, tenofovir, besifovir, and pegylated interferon), HCV (daclatasvir + asunaprevir, elbasvir/grazoprevir, glecaprevir/pibrentasvir, ledipasvir/sofosbuvir, ombitasvir/paritaprevir/ritonavir + dasabuvir, sofosbuvir, sofosbuvir + daclatasvir, ribavirin, and pegylated interferon), and HIV (abacavir, zidovudine, efavirenz, etravirine, nevirapine, rilpivirine, atazanavir, atazanavir–cobicistat, darunavir, darunavir–cobicistat, indinavir, lopinavir/ritonavir, nelfinavir, ritonavir, enfuvirtide, dolutegravir, raltegravir, abacavir–lamivudine (ABC/3TC), bictegravir–emtricitabine–tenofovir alafenamide (BIC/FTC/TAF), dolutegravir–abacavir–lamivudine (DTG/ABC/3TC), dolutegravir–lamivudine (DTG/3TC), doravirine–lamivudine–tenofovir disoproxil fumarate (DOR/3TC/TDF), elvitegravir–cobicistat–emtricitabine–tenofovir alafenamide (ECF/TAF or EVG/COBI/FTC/TAF), elvitegravir–cobicistat–emtricitabine–tenofovir disoproxil fumarate (ECF/TDF or EVG/COBI/FTC/TDF), tenofovir alafenamide–emtricitabine (TAF/FTC), tenofovir disoproxil fumarate–emtricitabine (TDF/FTC), zidovudine–lamivudine (ZDV/3TC), zidovudine–lamivudine (ZDV/3TC), and emtricitabine–rilpivirine hydrochloride–tenofovir disoproxil fumarate) (Supplementary Tables S1 and S2).

### 2.3. Statistical Analyses

Categorical variables were presented as frequencies and percentages, whereas continuous variables were expressed as mean values with standard deviations. A one-way ANOVA and the chi-square test were used to calculate differences between continuous and categorical variables among four groups (HBV monoinfection, HBV/HIV coinfection, HBV/HCV coinfection, and HBV/HIV/HCV coinfection). A modified CCI score, which excluded HIV infection, was used to avoid the overestimation of comorbidity burden in groups that included patients coinfected with HIV. Statistical significance was regarded at *p*-value < 0.05. All statistical analyses were performed using R version 3.5.1 (R Foundation for Statistical Computing).

## 3. Results

### 3.1. Demographics and Clinical Characteristics of Study Population

During the study period, the number of patients with hepatitis B virus (HBV) monoinfection ranged from 324,364 to 469,836 annually, with a corresponding prevalence of 0.79% to 1.06%. The number of patients with HBV/HIV coinfection ranged from 172 to 279 per year, with a prevalence of 0.00042% to 0.00069%. For HBV/HCV coinfection, the annual number of cases ranged from 2505 to 4503, corresponding to a prevalence of 0.0061% to 0.0103% (Table 1, Table S1). Among CHB patients, the prevalence of HIV coinfection was 0.053% to 0.066%, while that of HCV coinfection was 0.77% to 1.00% (Table S3).

**Figure 1:**
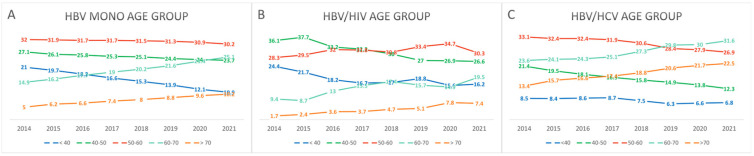
Temporal trends in age group distribution among patients with CHB, stratified by infection status ((**A**) HBV monoinfection, (**B**) HBV/HIV coinfection, (**C**) HBV/HCV coinfection) from 2014 to 2021. Age was categorized into five groups: 20–39, 40–49, 50–59, 60–69, and ≥70 years. The proportion of older individuals (≥60 years) gradually increased across all groups over time.

Using data from 2021 as an example, the proportion of male patients was significantly higher in the HBV/HIV coinfection group compared to the HBV monoinfection and HBV/HCV coinfection groups (HBV vs. HBV/HIV vs. HBV/HCV: 57% [267,688/469,834] vs. 90.6% [269/297] vs. 55% [2018/3672]; *p* < 0.001) (Table 1). The mean age was highest in the HBV/HCV coinfection group, followed by the HBV monoinfection and HBV/HIV coinfection groups (*p* < 0.001; Table 1). Over time, the proportion of individuals aged 60 years and older increased across all groups, indicating an overall aging trend (Figure 1, Table S3).

**Figure 2:**
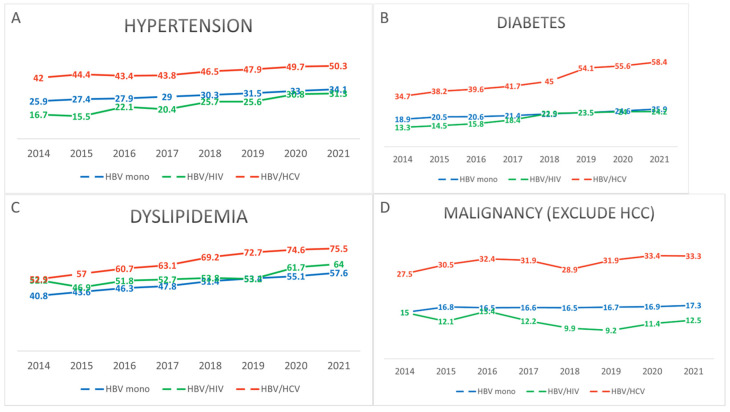
Temporal trends in the prevalence of comorbidities including (**A**) HTN, (**B**) DM, (**C**) DLD, and (**D**) malignancy (other than HCC) among patients with CHB, stratified by infection status (HBV monoinfection, HBV/HIV coinfection, HBV/HCV coinfection) from 2014 to 2021. A gradual increase in the prevalence of metabolic comorbidities was observed across all groups over time.

Regarding comorbidities, the prevalence of HTN and DM was highest in the HBV/HCV coinfection group, followed by the HBV monoinfection and HBV/HIV coinfection groups (HTN: 50.3% vs. 34.1% vs. 31.3%; diabetes mellitus: 58.4% vs. 25.9% vs. 24.2%, respectively; *p* < 0.001 for both). In contrast, the prevalence of DLD was higher in both the HBV/HCV and HBV/HIV coinfection groups compared to the HBV monoinfection group (75.5% vs. 64.0% vs. 57.6%, *p* < 0.001). These metabolic syndrome components showed an increasing trend over time across all groups (Figure 2, Table S3).

**Figure 3:**
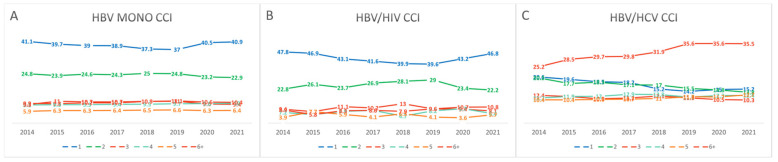
Temporal trends in the distribution of CCI scores among patients with CHB, stratified by infection status ((**A**) HBV monoinfection, (**B**) HBV/HIV coinfection, (**C**) HBV/HCV coinfection) from 2014 to 2021. The proportion of patients with higher CCI scores increased over time, particularly in the HBV/HCV coinfection group, indicating a growing comorbidity burden in this population.

The overall prevalence of malignancies other than HCC was significantly higher in the HBV/HCV coinfection group compared to the HBV monoinfection and HBV/HIV coinfection groups (33.3% vs. 17.3% vs. 12.5%, respectively; *p* < 0.001). The prevalence of a Charlson Comorbidity Index (CCI) score ≥ 2, excluding HIV infection, was also highest in the HBV/HCV coinfection group, followed by the HBV monoinfection and HBV/HIV coinfection groups (84.8% vs. 59.1% vs. 53.2%, *p* < 0.001). Throughout the study period, the proportion of patients with moderate-to-severe comorbidity (CCI ≥ 6, excluding HIV) was consistently the highest among those with HBV/HCV coinfection (Figure 3, Table S3).

### 3.2. Major Adverse Liver Outcomes of Study Population

**Figure 4:**
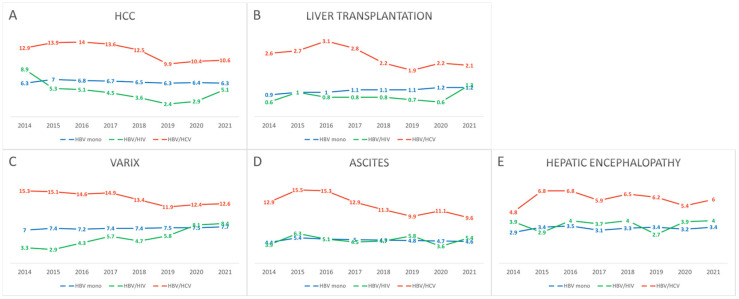
Temporal trends in the prevalence of MALO among patients with CHB, stratified by infection status (HBV monoinfection, HBV/HIV coinfection, HBV/HCV coinfection) from 2014 to 2021. The figure illustrates the annual proportions of patients diagnosed with (**A**) HCC, (**B**) liver transplantation, (**C**) varices, (**D**) ascites, and (**E**) hepatic encephalopathy.

Over the study period, the overall prevalence of major adverse liver outcomes (MALO) showed a declining trend across all groups. The prevalence of HCC was higher in the HBV/HCV coinfection group compared to the HBV monoinfection and HBV/HIV coinfection groups (6.3% vs. 5.1% vs. 10.6%, respectively; *p* < 0.001) (Table 2, Table S3). Although this trend remained relatively stable over time, the differences among the groups gradually narrowed (Figure S1). Age-stratified analysis showed that HCC occurrence increased or remained stable among individuals aged 60 years and older. Liver transplantation was also more common in the HBV/HCV coinfection group (1.2% vs. 1.3% vs. 2.1%, *p* < 0.001), with no substantial changes observed over time. The prevalence of cirrhosis-related complications decreased slightly in some intervals but remained relatively stable overall; however, it was consistently higher in the HBV/HCV coinfection group (Figure 4, Table S3).

### 3.3. Proportion of Antiviral Prescriptions, Including Nucleos(t)ide Analogues, in Study Population

**Figure 5:**
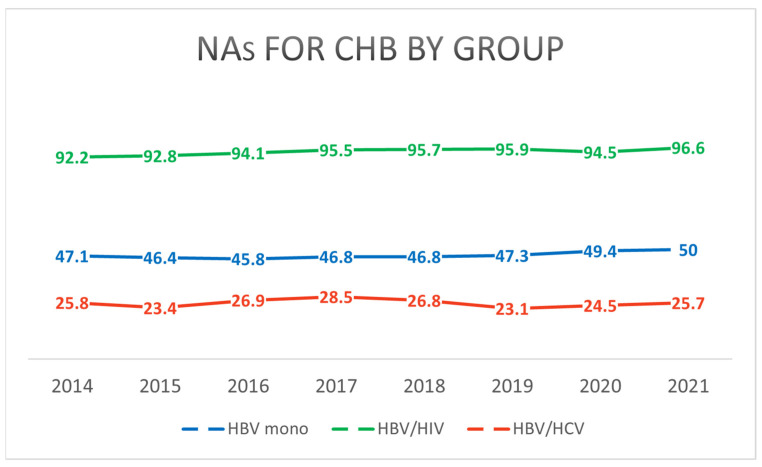
Annual trends in the proportion of patients receiving NAs for CHB from 2014 to 2021, stratified by infection group (HBV monoinfection, HBV/HIV coinfection, HBV/HCV coinfection). The figure shows the increasing uptake of NA prescriptions over time in all groups.

Interferon was not used as a treatment option beyond the early period of this study. The use of NAs for HBV was most frequent in the HBV/HIV coinfection group, with 96.6% (287/297) of patients receiving treatment, compared to 50.0% (234,685/469,834) in the HBV monoinfection group and 25.7% (945/3672) in the HBV/HCV coinfection group (*p* < 0.001) (Table 3, Table S3). Over time, the use of antiviral therapy for CHB showed a gradual increase across all groups (Figure 5, Table S3).

## 4. Discussion

This study utilized the HIRA database to investigate the national epidemiology of CHB patients with HIV or HCV coinfection. While limited studies have explored viral coinfections among individuals with CHB, this research offers valuable insight into the prevalence and characteristics of rare coinfections (HBV/HIV, HBV/HCV, HBV/HIV/HCV) based on a large-scale nationwide cohort. Given the challenges related to gaining access to sensitive HIV data, this study contributes meaningful data on a high-risk group that has rarely been evaluated using institutional cohorts with smaller sample sizes.

We found a gradual aging trend among CHB patients, likely attributable to population aging and the long-term administration of potent oral antivirals [20]. This pattern was also observed in coinfected populations. The number of patients and use of antiviral therapy increased over time, potentially due to guideline updates and expanded reimbursement criteria [21]. The overall HBV prevalence appeared slightly lower than that reported in other sources, possibly due to the use of operational definitions based on claims data limited to individuals who had received clinical care, in contrast with national surveillance data [22,23].

The HBV/HCV coinfection group showed a higher mean age and more frequent comorbidities, including HTN, DM, DLD, and higher CCI scores. Similar patterns were noted in patients with triple infection. The higher burden of metabolic disorders may be due not only to aging but also to persistent lipid metabolism disturbances even after HCV clearance [24,25]. These patients also showed a higher prevalence of MALO. Prior studies reported more advanced fibrosis and increased risk of decompensation in coinfected patients [26]. A 10-year Taiwanese cohort by Yang et al. found that ALT levels and HCC risk were significantly higher in HBV/HCV coinfection compared to HBV monoinfection [27], highlighting the need for close monitoring in this high-risk population [6].

A higher proportion of male patients was observed in the HBV/HIV coinfection group compared to other groups, which is consistent with the known male predominance in HIV infection [9]. Future studies should consider sex-stratified analysis when evaluating outcomes in coinfected populations [28,29]. Additionally, DLD was more prevalent in this group, potentially reflecting the effects of the virus and ART [30].

The prevalence of MALO was highest in the HBV/HCV group, followed by HBV monoinfection and HBV/HIV coinfection. This contradicts previous reports suggesting worse outcomes among patients coinfected with HBV/HIV compared to those with HBV monoinfection [31], warranting further adjustment for confounding variables such as age. Although HIV/AIDS is heavily weighted in the CCI, recent advances in ART have greatly improved prognosis, potentially leading to the overestimation of clinical severity in people living with HIV [32,33]. As HIV-positive patients now have longer life expectancies, the integrated management of comorbid conditions is essential [34].

Notably, antiviral therapy was underutilized in patients coinfected with HCV. This may reflect limitations in claims-based data, as out-of-pocket prescriptions are not captured [17]. Given accumulating evidence supporting prophylactic HBV therapy during DAA treatment in individuals infected with HCV [21,35], the prescription rate is expected to increase.

This study has several limitations that should be considered when interpreting the findings. First, although we utilized a cross-sectional design to capture annual trends in HBV coinfection with HIV and HCV among patients with CHB, this study was not able to statistically assess temporal associations or causal relationships between variables. Moreover, we could not analyze differences in patient management or medication regimens according to the treating specialty, such as between hepatology and infectious disease specialists in HBV/HIV coinfected patients. Second, as the analysis was based on the claims data provided by the HIRA, individuals who did not seek medical care were not included. Consequently, the estimated prevalence may differ from that reported in the national infectious disease notification system [23]. And patients were classified based on the presence of diagnostic codes regardless of antiviral treatment status. Therefore, individuals with HCV who achieved sustained virological response following DAA therapy were not excluded, which may lead to heterogeneity in the coinfection group. Third, while the Korean NHI system provides near-universal coverage, the dataset does not capture all medication usage. For instance, DAAs for HCV became reimbursable in 2014, and tenofovir alafenamide became covered starting in 2017. Therefore, prescriptions made outside the reimbursement framework were not reflected in the analysis. This limitation may result in discrepancies when comparing treatment patterns with studies conducted in other countries. Finally, although our findings provide novel insights into rare HBV coinfections, the small number of patients in the HBV/HIV and HBV/HIV/HCV groups necessitates cautious interpretation. The limited sample size may have reduced the statistical power and introduced variability in the estimates. Given the rarity and clinical sensitivity of these conditions, further studies with larger cohorts or multinational data may be required to confirm these observations and improve generalizability.

## 5. Conclusions

Using a nationally representative claims database, this study offers valuable insights into the real-world epidemiology of HIV and HCV coinfections among patients with CHB. By incorporating sensitive data, our findings provide a comprehensive understanding of coinfection patterns that may influence clinical outcomes and inform healthcare decision-making in routine practice. The observed heterogeneity in demographic and clinical characteristics among monoinfected and coinfected populations underscores the need for tailored management strategies. Given the shared transmission routes and increased disease burden associated with coinfections, differentiated approaches to screening, treatment, and long-term follow-up are warranted.

## Figures and Tables

**Table 1:** Descriptive analysis of baseline characteristics and non-liver comorbidities of chronic hepatitis B patients and HCV or HIV coinfection patients (2021).

Variable	HBV Monoinfection (*n* = 469,834)	HBV/HIV Coinfection (*n* = 297)	HBV/HCV Coinfection (*n* = 3672)	HBV/HCV/HIV Coinfection (*n* = 8)	*p* for Trend *
Male (%)	267,688 (57.0)	269 (90.6)	2018 (55.0)	6 (75.0)	<0.001
Age group, *n* (%)					
20–39	50,999 (10.9)	48 (16.2)	248 (6.8)	1 (12.5)	<0.001
40–49	111,242 (23.7)	79 (26.6)	450 (12.3)	4 (50.0)	
50–59	142,101 (30.2)	90 (30.3)	986 (26.9)	1 (12.5)	
60–69	117,735 (25.1)	58 (19.5)	1162 (31.6)	1 (12.5)	
70-	47,757 (10.2)	22 (7.4)	826 (22.5)	1 (12.5)	
HTN (%)	160,321 (34.1)	93 (31.3)	1847 (50.3)	2 (25.0)	<0.001
DM (%)	121,845 (25.9)	72 (24.2)	2143 (58.4)	2 (25.0)	<0.001
DLD (%)	270,583 (57.6)	190 (64.0)	2772 (75.5)	3 (37.5)	<0.001
Other malignancy (%)	81,055 (17.3)	37 (12.5)	1223 (33.3)	3 (37.5)	<0.001
CCI without HIV (%)					
1	191,964 (40.9)	139 (46.8)	559 (15.2)	3 (37.5)	<0.001
≥2	277,870 (59.1)	158 (53.2)	3113 (84.8)	5 (62.5)	
Precise_CCI (%) †					
1	191,964 (40.9)	139 (46.8)	559 (15.2)	3 (37.5)	<0.001
2	107,423 (22.9)	66 (22.2)	506 (13.8)	0 (0.0)	
3	44,205 (9.4)	24 (8.1)	380 (10.3)	0 (0.0)	
4	47,193 (10.0)	19 (6.4)	457 (12.4)	3 (37.5)	
5	29,957 (6.4)	17 (5.7)	467 (12.7)	1 (12.5)	
6	49,092 (10.4)	32 (10.8)	1303 (35.5)	1 (12.5)	

HTN, hypertension; DM, diabetes; DLD, dyslipidemia; † Deyo–Charlson Comorbidity Index was calculated excluding HIV disease score; * *p*-values were calculated from one-way ANOVA with repeated measures for continuous variables and generalized estimating equations for categorical variables between HBV monoinfection and matched controls.

**Table 2:** Descriptive analysis of major adverse liver outcomes among CHB patients and HCV or HIV coinfection patients (2021).

Variable	HBV Monoinfection (*n* = 469,834)	HBV/HIV Coinfection (*n* = 297)	HBV/HCV Coinfection (*n* = 3672)	HBV/HCV/HIV Coinfection (*n* = 8)	*p* for Trend *
HCC (%)	29,410 (6.3)	15 (5.1)	388 (10.6)	1 (12.5)	<0.001
Liver transplantation (%)	5658 (1.2)	4 (1.3)	77 (2.1)	0 (0.0)	<0.001
Varices (%)	35,989 (7.7)	25 (8.4)	461 (12.6)	0 (0.0)	<0.001
Variceal_hemorrhage (%)	432 (0.1)	0 (0.0)	8 (0.2)	0 (0.0)	0.089
Ascites (%)	21,748 (4.6)	16 (5.4)	354 (9.6)	1 (12.5)	<0.001
Hepatic_encephalopathy (%)	5961 (3.4)	12 (4.0)	219 (6.0)	1 (12.5)	<0.001
Hepatorenal_syndrome (%)	170 (0.0)	0 (0.0)	2 (0.1)	0 (0.0)	0.930
Spontaneous_bacterial_peritonitis (%)	173 (0.0)	0 (0.0)	3 (0.1)	0 (0.0)	0.554

* *p*-values were calculated from one-way ANOVA with repeated measures for continuous variables and generalized estimating equations for categorical variables between HBV monoinfection and matched controls.

**Table 3:** Descriptive analysis of antiviral agent utilization among CHB patients and HCV or HIV coinfection patients (2021).

Variable	HBV Monoinfection (*n* = 469,834)	HBV/HIV Coinfection (*n* = 297)	HBV/HCV Coinfection (*n* = 3672)	HBV/HCV/HIV Coinfection (*n* = 8)	*p* for Trend *
HBV_IFN (%)	0 (0.0)	0 (0.0)	0 (0.0)	0 (0.0)	NA
HBV_NAs (%)	234,685 (50.0)	287 (96.6)	945 (25.7)	7 (87.5)	<0.001
HIV_ART (%)	0 (0.0)	293 (98.7)	0 (0.0)	7 (87.5)	<0.001
HCV_IFN (%)	0 (0.0)	0 (0.0)	0 (0.0)	0 (0.0)	NA
HCV_DAA (%)	0 (0.0)	0 (0.0)	164 (4.5)	0 (0.0)	<0.001

IFN, interferon; NAs, nucleos(t)ide analogues; ART, antiretroviral therapy; DAA, direct acting antiviral agent; * *p*-values were calculated from one-way ANOVA with repeated measures for continuous variables and generalized estimating equations for categorical variables between CHB and matched controls.

## Data Availability

Raw data were generated at Health Insurance and Review Assessment in South Korea. Derived data supporting the findings of this study are available on request from corresponding author, Jae Yoon Jeong, on request.

## Supplementary Materials

The following supporting information can be downloaded at: https://www.mdpi.com/article/10.3390/pathogens14070715/s1, Supplement Figure S1. Annual trends in the age distribution of patients with HCC among individuals with CHB, stratified by infection group from 2014 to 2021; Supplement Table S1. Detail codes of comorbidities and medications; Supplement Table S2. Charlson comorbidity index score; Supplement Table S3. Annual trends in all clinical variables of study population with chronic hepatitis B (CHB), stratified by infection group (A. HBV monoinfection, B. HBV/HCV coinfection) from 2014 to 2021.

## Abbreviations

The following abbreviations are used in this manuscript:

WHO	World Health Organization
CHB	Chronic hepatitis B
HBV	Hepatitis B virus
HCV	Hepatitis C virus
HIVs	Human immunodeficiency viruses
ART	Antiretroviral therapy
DAA	Direct-acting antivirals
NAs	nucle-os(t)ide analogues
HCC	Hepatocellular carcinoma
HIRA	Health Insurance Review and Assessment Service
NHI	National Health Insurance
ICD-10	International Statistical Classification of Diseases and Related Health Problems, Tenth Revision
HTN	Hypertension
DM	Diabetes mellitus
DLD	Dyslipidemia
CCI	Charlson Comorbidity Index
MALOs	Major adverse liver outcomes
