# Supplementary material for: Epidemiologic Characteristics of Chronic Hepatitis B and Coinfections with Hepatitis C Virus or Human Immunodeficiency Virus in South Korea: A Nationwide Claims-Based Study Using the Korean Health Insurance Review and Assessment Service Database
Source: Pathogens. 2025 Jul 19;14(7):715. doi: 10.3390/pathogens14070715 (PMC12299982; doi:10.3390/pathogens14070715)
Supplement: Supplementary file 1 [file pathogens-14-00715-s001.zip › pathogens-3760095-supplementary.pdf]

Supplement figure S1. Annual trends in the age distribution of patients with HCC among individuals with chronic hepatitis B (CHB), stratified by infection group (A. HBV monoinfection, B. HBV/HCV coinfection) from 2014 to 2021

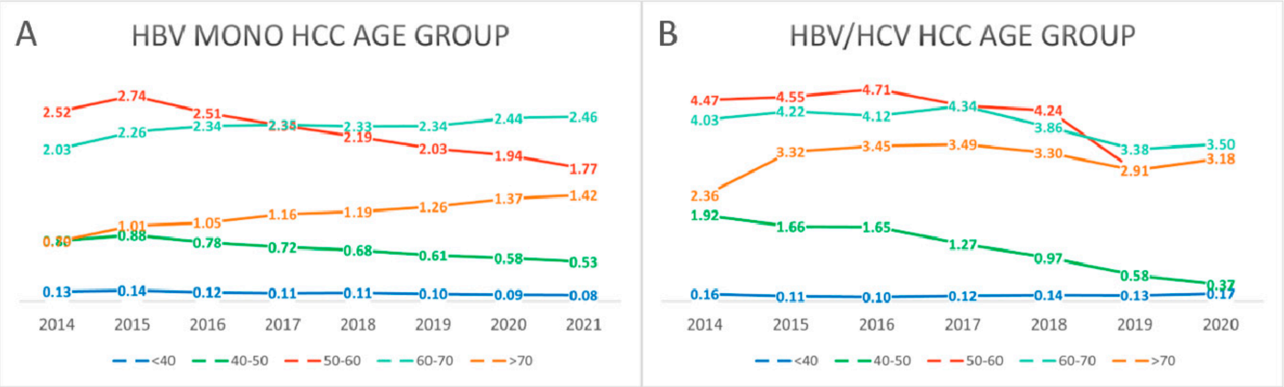

Supplement Table S1. Detail codes of comorbidities and medications

A. Detail codes of comorbidities

	**Disease code**	**Descriptions (ICD 10 code)**
**Cirrhosis**	K70.3	Alcoholic cirrhosis of liver
	K71.7	Toxic liver disease with fibrosis and cirrhosis of liver
	K72.1	Chronic hepatic failure
	K72.9	Hepatic failure, unspecified
	K74.5	Biliary cirrhosis, unspecified
	K74.6	Other and unspecified cirrhosis of liver
	K76.1	Cardiac cirrhosis (so-called) of liver
	K76.6	Portal hypertension
**major adverse liver outcomes :** Case where corresponds to at least 1 situation from the list	**Disease code**	**Descriptions (ICD 10 code)**
	I85.0	Oesophageal varices with bleeding
	I85.9	Oesophageal varices without bleeding / Oesophageal varices NOS
	I98.2	Oesophageal varices without bleeding in diseases classified elsewhere
	I98.3	Oesophageal varices with bleeding in diseases classified elsewhere
	I86.4	Gastric varices
	R18	Ascites
	G93.4	Encephalopathy, unspecified
	G31.2	Degeneration of nervous system due to alcohol
	K72.01	Acute and subacute hepatic failure, with coma

	K72.11	Chronic hepatic failure, with coma
	K72.91	Hepatic failure, unspecified, with coma
	K76.7	Hepatorenal syndrome
	K65.0	Acute peritonitis
	K65.9	Peritonitis, unspecified
	**code**	**Descriptions (medical cost code)**
	Q7631	Endoscopic Sclerotherapy
	Q7632	Endoscopic Sclerotherapy
	Q7633	Endoscopic variceal ligation
	Q7634	Endoscopic variceal ligation
	C8050	Paracentesis, Abdominal Paracentesis
	C8051	Paracentesis, Abdominal Paracentesis
	**varices**	
	Patients who have at least 1 or more medical record by year via code I.	
	Patients who have at least 1 or more medical record by year via code I & code K.	
	Patients who were prescribed with B-blocker along with code K	
	**Ascites**	
	Patients who have at least 1 or more medical record by year via R18 & code K.	
	Patients who were prescribed with Diuretic along with code K	
	Patients who were prescribed with "Albumin" along with code K and "Paracentesis, Abdominal Paracentesis"	
	Kidney	
	Patient who have at least 1 or more medical record by year via K767	
	Patient who was prescribed with Terlipressin & Albumin for more than 4 days	
	**infection**	
	Patient that have at least 1 or more medical record by year via K658, 659, code K, and "Paracentesis, Abdominal Paracentesis" simultaneously	
	Encephalopathy	
	Patients who were prescribed with Duphalac along with code K	
	Patient who was prescribed with Duphalac along with enema procedure	
**Hypertension**	**Disease code**	**Descriptions (ICD 10 code)**
	I10	Essential (primary) hypertension
**Diabetes**	**Disease code**	**Descriptions (ICD 10 code)**
	E10	Type 1 diabetes mellitus
	E11	Type 2 diabetes mellitus
	E12	Malnutrition-related diabetes mellitus
	E13	Other specified diabetes mellitus
	E14	Unspecified diabetes mellitus
**Dyslipidemia**	**Disease code**	**Descriptions (ICD 10 code)**
	E78	Disorders of lipoprotein metabolism and other lipidemias

**Malignant neoplasms**	**RID code**	**Descriptions (RID code)**
	V027	Specific day when unregistered cancer patients received consultation regards to corresponding disease (C00 ~ C97, D00 ~ D09, D32 ~ D33, D37 ~ D48)
	V193	「Standard regards to partial individual expense assessment」 [Enclosure] In case when registered cancer patients, according to the fixed form, receive consultation regards to the corresponding disease during the 5 years from the registration date (C00 ~ C97, D00 ~ D09, D32 ~ D33, D37 ~ D48)
	**Disease code**	**Descriptions (KCD 7 code)**
	C22	Malignant neoplasm of liver and intrahepatic bile ducts
	**Disease code**	**Descriptions (KCD 7 code)**
	C00 - C14	Malignant neoplasms of lip, oral cavity and pharynx
	C15	Malignant neoplasm of esophagus
	C16	Malignant neoplasm of stomach
	C18 - C21	Malignant neoplasms of colon, rectum and anus
	C22	Malignant neoplasms of liver / intrahepatic bile ducts
	C25	Malignant neoplasm of pancreas
	C32	Malignant neoplasm of larynx
	C33 - C34	Malignant neoplasms of trachea, bronchus and lung
	C43	Malignant melanoma of skin
	C50	Malignant neoplasm of breast
	C53	Malignant neoplasm of cervix uteri
	C54 - C55	Malignant neoplasms of corpus uteri and uterus, part unspecified
	C56	Malignant neoplasm of ovary
	C61	Malignant neoplasm of prostate
	C64 - C65	Malignant neoplasms of kidney and renal pelvis
	C67	Malignant neoplasm of bladder
	C70 - C72	Malignant neoplasms of meninges, brain and other parts of central nervous system
	C81	Hodgkin's disease
	C82 - C85	Non-Hodgkin's lymphoma
	C91 - C95	Leukemia
	C88, C90	Multiple myeloma and immunoproliferative neoplasms
	C96	Other and unspecified malignant neoplasms of lymphoid, hematopoietic & related tissue
	C00-C97	All other and unspecified malignant neoplasms
**Liver transplant:** Liver transplant cannot be defined by ICD-10 code, but the code of medical cost can be used in this case.	**code**	**Descriptions (medical cost code)**
	Q8040	Liver Transplantation from Cadaver Donor-Total
	Q8041	Liver Transplantation from Cadaver Donor
	Q8042	Liver Transplantation from Cadaver Donor
	Q8043	Liver Transplantation from Cadaver Donor-Split-Left Lateral Segment
	Q8044	Liver Transplantation from Cadaver Donor-Split-Left Lobe

	Q8045	Partial Liver Transplantation from Living Donor-Left Lateral Segment
	Q8046	Partial Liver Transplantation from Living Donor-Left Lobe
	Q8047	Partial Liver Transplantation from Living Donor-Right Lobe
	Q8048	Partial Liver Transplantation from Living Donor-Extended Right Lobe
	Q8049	Partial Liver Transplantation from Living Donor-Modified Right Lobe
	Q8050	Partial Liver Transplantation from Living Donor-Dual Graft
	Q8140	Liver Retransplantation
	Q8141	Liver Retransplantation
	Q8142	Liver Retransplantation
	Q8143	Liver Retransplantation
	Q8144	Liver Retransplantation
	Q8145	Liver Retransplantation
	Q8146	Liver Retransplantation
	Q8147	Liver Retransplantation
	Q8148	Liver Retransplantation
	Q8149	Liver Retransplantation
	Q8150	Liver Retransplantation

B. Detail codes of medications

	**Drug Code**	**Descriptions (Molecule incl. volume)**
**nucleos(t)ide analogues**	180901ATB	lamivudine 0.1g
	180901ASY	lamivudine 100mg
	457501ATB	adefovir dipivoxil 10mg
	487802ACH	clevudine 30mg
	487801ACH	clevudine 10mg
	506001ATB	telbivudine 0.6g
	487202ATB	entecavir 0.5mg
	487202ATD	entecavir 0.5mg
	487203ATB	entecavir 1mg
	487203ATD	entecavir 1mg
	493901ATB	tenofovir disoproxil fumarate 0.3g (as tenofovir disoproxil 0.245g)
	664901ATB	tenofovir disoproxil 0.245g
	665001ATB	tenofovir disoproxil phosphate (as tenofovir disoproxil 0.245g)
	665101ATB	tenofovir disoproxil asparate (as tenofovir disoproxil 0.245g)
	665201ATB	tenofovir disoproxil orotate (as tenofovir disoproxil 0.245g)
	665501ATB	tenofovir disoproxil hemiedisylate (as tenofovir disoproxil 0.245g)
	665301ATB	tenofovir alafenamide fumarate (as tenofovir alafenamide 25mg)
	665401ATB	besifovir dipivoxil maleate (as besifovir dipivoxil 0.15g)

	Drug Code	Descriptions (Molecule incl. volume)
direct-acting antivirals	638101ATB	daclatasvir hydrochloride (as daclatasvir 60mg)
	638001ACH	"asunaprevir 0.1g"
	644401ATB	sofosbuvir 0.4g
	645800ATB	ledipasvir 90mg /sofosbuvir 0.4g
	657000ATB	Elbasvir 50mg / Grazoprevir 0.1g
	658600ATB	Ombitasvir 12.5mg / Paritaprevir 75mg / Ritonavir 50mg
	658701ATB	Dasabuvir 0.25
	669700ATB	Glecaprevir 0.1g / Pibrentasvir 40mg

	Drug Code	Descriptions (Molecule incl. volume)	years
antiretroviral therapy	420101ATB	Abacavir 0.3g	2009-2020
	180901ATB	Lamivudine 100mg	2008-2020
	180902ATB	Lamivudine 150mg	2009-2019
	180930ASY	lamivudine 1.2g 시럽	2015-2017
	665301ATB	Tenofovir alafenamide 25mg	2017-2020
	493901ATB	tenofovir disoproxil fumarate 0.3g	2012-2020
	664901ATB	tenofovir disoproxil 0.245g	2017-2020
	665001ATB	tenofovir disoproxil phosphate	2017-2020
	665101ATB	tenofovir disoproxil asparate	2017-2020
	665201ATB	tenofovir disoproxil orotate	2017-2020
	665501ATB	tenofovir disoproxil hemiedisylate	2017-2020
	250201ACH	Zidovudine 100mg	2008-2020
	151002ATB	Efavirenz 600mg	2008-2020
	508901ATB	Etravirine 0.1g	2010-2020
	200801ATB	Nevirapine 200mg	2008-2020
	617801ATB	Rilpivirine 25mg	2013-2020
	458502ACH	Atazanavir 150mg	2008-2020
	458503ACH	Atazanavir 200mg	2008-2020
	646700ATB	Atazanavir-cobicistat	2016-2020
	498702ATB	Darunavir 0.6g	2011-2020
	498703ATB	Darunavir 0.4g	2011-2020
	647600ATB	Darunavir-cobicistat	2016-2020
	174601ACH	Indinavir 250mg	2008-2017
	174603ACH	Indinavir 500mg	2008-2017
	486300ATB	Lopinavir/ritonavir(20/50)	2008-2020
	516600ATB	Lopinavir/ritonavir(100/25)	2011-2015
	200102APD	Nelfinavir 50mg	2008-2013
	200101ATB	Nelfinavir 250mg	2008-2019
	224401ACH	Ritonavir 100mg(캅셀)	2008-2020

	224401ACS	Ritonavir 100mg(연질캅셀)	2008-2016
	224401ATB	Ritonavir 100mg(정)	2015-2020
	224430ALQ	Ritonavir 19.2g(액)	2015-2020
	461401BIJ	Enfuvirtide 108mg	2008-2020
	628601ATB	Dolutegravir 50mg	2016-2020
	506301ATB	Raltegravir 0.4g	2010-2020
	506302ATB	Raltegravir 0.6g	2018-2020
	517300ATB	Abacavir-lamivudine (ABC/3TC)	2011-2020
	676800ATB	Bictegravir-emtricitabine-tenofovir alafenamide (BIC/FTC/TAF)	2019-2020
	641600ATB	Dolutegravir-abacavir-lamivudine (DTG/ABC/3TC)	2015-2020
	687400ATB	Dolutegravir-lamivudine (DTG/3TC)	2020
		Doravirine-lamivudine-tenofovir disoproxil fumarate (DOR/3TC/TDF)	
	655900ATB	Elvitegravir-cobicistat-emtricitabine-tenofovir alafenamide (ECF/TAF or EVG/COBI/FTC/TAF)	2017-2020
	623400ATB	Elvitegravir-cobicistat-emtricitabine-tenofovir disoproxil fumarate (ECF/TDF or EVG/COBI/FTC/TDF)	2015-2018
	658300ATB	Tenofovir alafenamide-emtricitabine (TAF/FTC) (10mg/0.2g)	2017-2020
	658400ATB	Tenofovir alafenamide-emtricitabine (TAF/FTC) (25mg/0.2g)	2017-2020
	599900ATB	Tenofovir disoproxil fumarate-emtricitabine (TDF/FTC)	2012-2020
	430700ATB	Zidovudine-lamivudine (ZDV/3TC)	2008-2010
	513100ATB	Zidovudine-lamivudine (ZDV/3TC) (0.3g/0.15g)	2011-2020
	248100ATB	Emtricitabine-rilpivirine hydrochloride-tenofovir disoproxil fumarate	2015-2018

	**Drug Code**	**Descriptions (Molecule incl. volume)**
**Interferon**	452631BIJ	peg interferon α-2a 90μg(0.18mg/mL)
	452602BIJ	peg interferon α-2a 0.135mg(0.27mg/mL)
	452630BIJ	peg interferon α-2a(40kD) 0.18mg(0.36mg/mL)
	454831BIJ	peg interferon α-2b 80μg(0.16mg/mL)
	454830BIJ	peg interferon α-2b 50μg(0.1mg/mL)
	454833BIJ	peg interferon α-2b 0.12mg(0.24mg/mL)
	454832BIJ	peg interferon α-2b 0.1mg(0.2mg/mL)
	454834BIJ	peg interferon α-2b 0.15mg(0.3mg/mL)

	**Drug Code**	**Descriptions (Molecule incl. volume)**
**Comedication for MALO**	219901ATB	propranolol HCl 10mg
	219904ATB	propranolol HCl 40mg
	219906ACR	propranolol HCl 80mg
	219905ACR	propranolol HCl 160mg
	198301ATB	nadolol 40mg

	125003ATB	carvedilol 6.25mg
	125001ATB	carvedilol 12.5mg
	125002ATB	carvedilol 25mg
	125005ATB	carvedilol 3.125mg
	125004ACR	carvedilol 64mg
	125006ACR	carvedilol 32mg
	125007ACR	carvedilol 16mg
	125008ACR	carvedilol 8mg
	662201ATB	S-carvedilol 6.25mg
	662202ATB	S-carvedilol 12.5mg
	163801ATB	furosemide 40mg
	242002ATB	torasemide 2.5mg
	242003ATB	torasemide 5mg
	242001ATB	torasemide 10mg
	242004ATB	torasemide 20mg
	231101ATB	spironolactone 25mg
	231102ATB	spironolactone 50mg
	106901ATB	amiloride HCl 5mg
	452001ATB	rifaximin 200mg

*Note: RID: registration codes from the rare/intractable diseases patient-support program

*Note: Sub-codes for above disease and drug codes were also included for analysis

Supplement Table S2. Charlson comorbidity index score

Disease	ICD-10 codes	CCI score
**Diabetes mellitus**	E10–E14	1
**Myocardial infarction**	I21, I22, I25	1
**Congestive cardiac failure**	I50	1
**Peripheral vascular disease**	I70–I79	1
**Cerebrovascular disease**	I60–I69	1
**Dementia**	F03, G30	1
**Chronic pulmonary disease**	J41, J42, J43, J44, J45, J47, J64	1
**Rheumatological disease**	M30–M36, M06	1
**Gastric or peptic ulcer**	K25, K26	1
**Mild liver disease**	B18, B19, K70–K77	1
**Hemiplegia or paraplegia**	G80, G81, G82	2
**Moderate or severe renal disease**	N17–N19	2
**Any malignancy, including lymphoma and leukemia, except basal cell cancer of skin**	C00–C41, C43, C45–C72, C74, C75, C81–C96	2
**Metastatic solid tumour**	C76–C80	6
**AIDS/HIV infection**	B20-B24	6

Supplement Table S3. Charlson comorbidity index score

Variable_2014	level	HBV mono	HBV/HIV	HBV/HCV	HBV/HCV/HIV	p
n		324355	180	2505	1	
Sex (%)	1	193855 (59.8)	165 (91.7)	1511 (60.3)	1 (100.0)	<0.001
	2	130500 (40.2)	15 (8.3)	994 (39.7)	0 (0.0)	
Age (%)	40	68272 (21.0)	44 (24.4)	212 (8.5)	0 (0.0)	<0.001
	40-50	87785 (27.1)	65 (36.1)	537 (21.4)	1 (100.0)	
	50-60	103652 (32.0)	51 (28.3)	829 (33.1)	0 (0.0)	
	60-70	48348 (14.9)	17 (9.4)	592 (23.6)	0 (0.0)	
	70-100	16298 (5.0)	3 (1.7)	335 (13.4)	0 (0.0)	
HCC (%)	0	304001 (93.7)	164 (91.1)	2181 (87.1)	1 (100.0)	<0.001
	1	20354 (6.3)	16 (8.9)	324 (12.9)	0 (0.0)	
Liver transplantation (%)	0	321339 (99.1)	179 (99.4)	2440 (97.4)	1 (100.0)	<0.001
	1	3016 (0.9)	1 (0.6)	65 (2.6)	0 (0.0)	
HTN (%)	0	240429 (74.1)	150 (83.3)	1454 (58.0)	1 (100.0)	<0.001
	1	83926 (25.9)	30 (16.7)	1051 (42.0)	0 (0.0)	
DM (%)	0	262973 (81.1)	156 (86.7)	1636 (65.3)	1 (100.0)	<0.001
	1	61382 (18.9)	24 (13.3)	869 (34.7)	0 (0.0)	
DLD (%)	0	192151 (59.2)	86 (47.8)	1180 (47.1)	1 (100.0)	<0.001
	1	132204 (40.8)	94 (52.2)	1325 (52.9)	0 (0.0)	
Malignancy (%)	0	275583 (85.0)	153 (85.0)	1817 (72.5)	1 (100.0)	<0.001
	1	48772 (15.0)	27 (15.0)	688 (27.5)	0 (0.0)	
Varices (%)	0	301707 (93.0)	174 (96.7)	2122 (84.7)	1 (100.0)	<0.001
	1	22648 (7.0)	6 (3.3)	383 (15.3)	0 (0.0)	
Variceal_hemorrhage (%)	0	323900 (99.9)	179 (99.4)	2495 (99.6)	1 (100.0)	0.003
	1	455 (0.1)	1 (0.6)	10 (0.4)	0 (0.0)	

Ascites (%)	0	310015 (95.6)	173 (96.1)	2183 (87.1)	1 (100.0)	<0.001
	1	14340 (4.4)	7 (3.9)	322 (12.9)	0 (0.0)	
Hepatic_encephalopathy (%)	0	314811 (97.1)	173 (96.1)	2385 (95.2)	1 (100.0)	<0.001
	1	9544 (2.9)	7 (3.9)	120 (4.8)	0 (0.0)	
Hepatorenal_syndrome (%)	0	324311 (100.0)	180 (100.0)	2504 (100.0)	1 (100.0)	0.734
	1	44 (0.0)	0 (0.0)	1 (0.0)	0 (0.0)	
Spontaneous_bacterial_peritonitis(%)	0	324039 (99.9)	180 (100.0)	2499 (99.8)	1 (100.0)	0.152
	1	316 (0.1)	0 (0.0)	6 (0.2)	0 (0.0)	
HBV_antivirus_IFN (%)	0	324331 (100.0)	180 (100.0)	2465 (98.4)	1 (100.0)	<0.001
	1	24 (0.0)	0 (0.0)	40 (1.6)	0 (0.0)	
HBV_antivirus_Oral (%)	0	171740 (52.9)	14 (7.8)	1859 (74.2)	0 (0.0)	<0.001
	1	152615 (47.1)	166 (92.2)	646 (25.8)	1 (100.0)	
HIV_antivirus (%)	0	324355 (100.0)	13 (7.2)	2505 (100.0)	0 (0.0)	<0.001
	1	0 (0.0)	167 (92.8)	0 (0.0)	1 (100.0)	
HCV_antivirus_IFN (%)	0	324331 (100.0)	180 (100.0)	2465 (98.4)	1 (100.0)	<0.001
	1	24 (0.0)	0 (0.0)	40 (1.6)	0 (0.0)	
HCV_antivirus_Oral (%)	0	324355 (100.0)	180 (100.0)	2505 (100.0)	1 (100.0)	NA
	1	0 (0.0)	0 (0.0)	0 (0.0)	0 (0.0)	
CCI_wo_HIV (%)	1	133245 (41.1)	86 (47.8)	517 (20.6)	0 (0.0)	<0.001
	2	191110 (58.9)	94 (52.2)	1988 (79.4)	1 (100.0)	
precise_CCI (%)	1	133245 (41.1)	86 (47.8)	517 (20.6)	0 (0.0)	<0.001
	2	80409 (24.8)	41 (22.8)	505 (20.2)	1 (100.0)	
	3	32132 (9.9)	17 (9.4)	310 (12.4)	0 (0.0)	
	4	29397 (9.1)	13 (7.2)	280 (11.2)	0 (0.0)	
	5	19166 (5.9)	7 (3.9)	261 (10.4)	0 (0.0)	
	6	30006 (9.3)	16 (8.9)	632 (25.2)	0 (0.0)	

Variable_2015	level	HBV mono	HBV/HIV	HBV/HCV	HBV/HCV/HIV	p
n		367982	207	3674	4	
Sex (%)	1	218793 (59.5)	189 (91.3)	2157 (58.7)	4 (100.0)	<0.001
	2	149189 (40.5)	18 (8.7)	1517 (41.3)	0 (0.0)	
Age (%)	40	72324 (19.7)	45 (21.7)	308 (8.4)	1 (25.0)	<0.001
	40-50	96199 (26.1)	78 (37.7)	716 (19.5)	0 (0.0)	
	50-60	117273 (31.9)	61 (29.5)	1189 (32.4)	2 (50.0)	
	60-70	59536 (16.2)	18 (8.7)	884 (24.1)	0 (0.0)	
	70-100	22650 (6.2)	5 (2.4)	577 (15.7)	1 (25.0)	
HCC (%)	0	342107 (93.0)	196 (94.7)	3165 (86.1)	4 (100.0)	<0.001
	1	25875 (7.0)	11 (5.3)	509 (13.9)	0 (0.0)	
Liver transplantation (%)	0	364412 (99.0)	205 (99.0)	3574 (97.3)	4 (100.0)	<0.001
	1	3570 (1.0)	2 (1.0)	100 (2.7)	0 (0.0)	
HTN (%)	0	267261 (72.6)	175 (84.5)	2042 (55.6)	2 (50.0)	<0.001
	1	100721 (27.4)	32 (15.5)	1632 (44.4)	2 (50.0)	
DM (%)	0	292444 (79.5)	177 (85.5)	2271 (61.8)	2 (50.0)	<0.001
	1	75538 (20.5)	30 (14.5)	1403 (38.2)	2 (50.0)	
DLD (%)	0	207665 (56.4)	110 (53.1)	1580 (43.0)	1 (25.0)	<0.001
	1	160317 (43.6)	97 (46.9)	2094 (57.0)	3 (75.0)	
Malignancy (%)	0	306294 (83.2)	182 (87.9)	2552 (69.5)	4 (100.0)	<0.001
	1	61688 (16.8)	25 (12.1)	1122 (30.5)	0 (0.0)	
Varices (%)	0	340744 (92.6)	201 (97.1)	3119 (84.9)	4 (100.0)	<0.001
	1	27238 (7.4)	6 (2.9)	555 (15.1)	0 (0.0)	
Variceal_hemorrhage (%)	0	367287 (99.8)	207 (100.0)	3666 (99.8)	4 (100.0)	0.905
	1	695 (0.2)	0 (0.0)	8 (0.2)	0 (0.0)	
Ascites (%)	0	348058 (94.6)	194 (93.7)	3106 (84.5)	3 (75.0)	<0.001

	1	19924 (5.4)	13 (6.3)	568 (15.5)	1 (25.0)	
Hepatic_encephalopathy (%)	0	355576 (96.6)	201 (97.1)	3425 (93.2)	4 (100.0)	<0.001
	1	12406 (3.4)	6 (2.9)	249 (6.8)	0 (0.0)	
Hepatorenal_syndrome (%)	0	367715 (99.9)	207 (100.0)	3668 (99.8)	4 (100.0)	0.237
	1	267 (0.1)	0 (0.0)	6 (0.2)	0 (0.0)	
Spontaneous_bacterial_peritonitis(%)	0	367642 (99.9)	207 (100.0)	3660 (99.6)	4 (100.0)	<0.001
	1	340 (0.1)	0 (0.0)	14 (0.4)	0 (0.0)	
HBV_antivirus_IFN (%)	0	367964 (100.0)	207 (100.0)	3632 (98.9)	4 (100.0)	<0.001
	1	18 (0.0)	0 (0.0)	42 (1.1)	0 (0.0)	
HBV_antivirus_Oral (%)	0	197270 (53.6)	15 (7.2)	2815 (76.6)	2 (50.0)	<0.001
	1	170712 (46.4)	192 (92.8)	859 (23.4)	2 (50.0)	
HIV_antivirus (%)	0	367982 (100.0)	11 (5.3)	3674 (100.0)	2 (50.0)	<0.001
	1	0 (0.0)	196 (94.7)	0 (0.0)	2 (50.0)	
HCV_antivirus_IFN (%)	0	367964 (100.0)	207 (100.0)	3632 (98.9)	4 (100.0)	<0.001
	1	18 (0.0)	0 (0.0)	42 (1.1)	0 (0.0)	
HCV_antivirus_Oral (%)	0	367982 (100.0)	207 (100.0)	3580 (97.4)	4 (100.0)	<0.001
	1	0 (0.0)	0 (0.0)	94 (2.6)	0 (0.0)	
CCI_wo_HIV (%)	1	146256 (39.7)	97 (46.9)	719 (19.6)	1 (25.0)	<0.001
	2	221726 (60.3)	110 (53.1)	2955 (80.4)	3 (75.0)	
precise_CCI (%)	1	146256 (39.7)	97 (46.9)	719 (19.6)	1 (25.0)	<0.001
	2	87913 (23.9)	54 (26.1)	652 (17.7)	1 (25.0)	
	3	36074 (9.8)	16 (7.7)	437 (11.9)	0 (0.0)	
	4	34225 (9.3)	12 (5.8)	437 (11.9)	0 (0.0)	
	5	23028 (6.3)	16 (7.7)	383 (10.4)	1 (25.0)	
	6	40486 (11.0)	12 (5.8)	1046 (28.5)	1 (25.0)	

Variable_2016	level	HBV mono	HBV/HIV	HBV/HCV	HBV/HCV/HIV	p
n		402996	253	4053	8	
Sex (%)	1	235330 (58.4)	234 (92.5)	2376 (58.6)	4 (50.0)	<0.001
	2	167666 (41.6)	19 (7.5)	1677 (41.4)	4 (50.0)	
Age (%)	40	73325 (18.2)	46 (18.2)	349 (8.6)	3 (37.5)	<0.001
	40-50	103778 (25.8)	84 (33.2)	733 (18.1)	1 (12.5)	
	50-60	127880 (31.7)	81 (32.0)	1314 (32.4)	3 (37.5)	
	60-70	71487 (17.7)	33 (13.0)	985 (24.3)	1 (12.5)	
	70-100	26526 (6.6)	9 (3.6)	672 (16.6)	0 (0.0)	
HCC (%)	0	375603 (93.2)	240 (94.9)	3484 (86.0)	8 (100.0)	<0.001
	1	27393 (6.8)	13 (5.1)	569 (14.0)	0 (0.0)	
Liver transplantation (%)	0	399003 (99.0)	251 (99.2)	3926 (96.9)	8 (100.0)	<0.001
	1	3993 (1.0)	2 (0.8)	127 (3.1)	0 (0.0)	
HTN (%)	0	290654 (72.1)	197 (77.9)	2292 (56.6)	7 (87.5)	<0.001
	1	112342 (27.9)	56 (22.1)	1761 (43.4)	1 (12.5)	
DM (%)	0	319888 (79.4)	213 (84.2)	2449 (60.4)	7 (87.5)	<0.001
	1	83108 (20.6)	40 (15.8)	1604 (39.6)	1 (12.5)	
DLD (%)	0	216502 (53.7)	122 (48.2)	1592 (39.3)	4 (50.0)	<0.001
	1	186494 (46.3)	131 (51.8)	2461 (60.7)	4 (50.0)	
Malignancy (%)	0	336314 (83.5)	214 (84.6)	2738 (67.6)	7 (87.5)	<0.001
	1	66682 (16.5)	39 (15.4)	1315 (32.4)	1 (12.5)	
Varices (%)	0	373997 (92.8)	242 (95.7)	3461 (85.4)	8 (100.0)	<0.001
	1	28999 (7.2)	11 (4.3)	592 (14.6)	0 (0.0)	
Variceal_hemorrhage (%)	0	402433 (99.9)	253 (100.0)	4039 (99.7)	8 (100.0)	0.006
	1	563 (0.1)	0 (0.0)	14 (0.3)	0 (0.0)	

Ascites (%)	0	382307 (94.9)	240 (94.9)	3431 (84.7)	8 (100.0)	<0.001
	1	20689 (5.1)	13 (5.1)	622 (15.3)	0 (0.0)	
Hepatic_encephalopathy (%)	0	389059 (96.5)	243 (96.0)	3776 (93.2)	7 (87.5)	<0.001
	1	13937 (3.5)	10 (4.0)	277 (6.8)	1 (12.5)	
Hepatorenal_syndrome (%)	0	402790 (99.9)	253 (100.0)	4048 (99.9)	8 (100.0)	0.243
	1	206 (0.1)	0 (0.0)	5 (0.1)	0 (0.0)	
Spontaneous_bacterial_peritonitis(%)	0	402683 (99.9)	252 (99.6)	4044 (99.8)	8 (100.0)	0.003
	1	313 (0.1)	1 (0.4)	9 (0.2)	0 (0.0)	
HBV_antivirus_IFN (%)	0	402909 (100.0)	253 (100.0)	4004 (98.8)	8 (100.0)	<0.001
	1	87 (0.0)	0 (0.0)	49 (1.2)	0 (0.0)	
HBV_antivirus_Oral (%)	0	218335 (54.2)	15 (5.9)	2964 (73.1)	2 (25.0)	<0.001
	1	184661 (45.8)	238 (94.1)	1089 (26.9)	6 (75.0)	
HIV_antivirus (%)	0	402996 (100.0)	12 (4.7)	4053 (100.0)	2 (25.0)	<0.001
	1	0 (0.0)	241 (95.3)	0 (0.0)	6 (75.0)	
HCV_antivirus_IFN (%)	0	402909 (100.0)	253 (100.0)	4004 (98.8)	8 (100.0)	<0.001
	1	87 (0.0)	0 (0.0)	49 (1.2)	0 (0.0)	
HCV_antivirus_Oral (%)	0	402996 (100.0)	253 (100.0)	3569 (88.1)	8 (100.0)	<0.001
	1	0 (0.0)	0 (0.0)	484 (11.9)	0 (0.0)	
CCI_wo_HIV (%)	1	157073 (39.0)	109 (43.1)	749 (18.5)	4 (50.0)	<0.001
	2	245923 (61.0)	144 (56.9)	3304 (81.5)	4 (50.0)	
precise_CCI (%)	1	157073 (39.0)	109 (43.1)	749 (18.5)	4 (50.0)	<0.001
	2	99273 (24.6)	60 (23.7)	740 (18.3)	2 (25.0)	
	3	40930 (10.2)	28 (11.1)	443 (10.9)	1 (12.5)	
	4	37512 (9.3)	20 (7.9)	488 (12.0)	0 (0.0)	
	5	25259 (6.3)	15 (5.9)	430 (10.6)	0 (0.0)	
	6	42949 (10.7)	21 (8.3)	1203 (29.7)	1 (12.5)	

Variable_2017	level	HBV mono	HBV/HIV	HBV/HCV	HBV/HCV/HIV	p
n		418639	245	4099	10	
Sex (%)	1	243785 (58.2)	231 (94.3)	2355 (57.5)	9 (90.0)	<0.001
	2	174854 (41.8)	14 (5.7)	1744 (42.5)	1 (10.0)	
Age (%)	40	69606 (16.6)	41 (16.7)	356 (8.7)	2 (20.0)	<0.001
	40-50	105925 (25.3)	79 (32.2)	691 (16.9)	3 (30.0)	
	50-60	132601 (31.7)	78 (31.8)	1307 (31.9)	3 (30.0)	
	60-70	79701 (19.0)	38 (15.5)	1030 (25.1)	1 (10.0)	
	70-100	30806 (7.4)	9 (3.7)	715 (17.4)	1 (10.0)	
HCC (%)	0	390653 (93.3)	234 (95.5)	3543 (86.4)	10 (100.0)	<0.001
	1	27986 (6.7)	11 (4.5)	556 (13.6)	0 (0.0)	
Liver transplantation (%)	0	414139 (98.9)	243 (99.2)	3985 (97.2)	10 (100.0)	<0.001
	1	4500 (1.1)	2 (0.8)	114 (2.8)	0 (0.0)	
HTN (%)	0	297437 (71.0)	195 (79.6)	2302 (56.2)	8 (80.0)	<0.001
	1	121202 (29.0)	50 (20.4)	1797 (43.8)	2 (20.0)	
DM (%)	0	329012 (78.6)	200 (81.6)	2390 (58.3)	8 (80.0)	<0.001
	1	89627 (21.4)	45 (18.4)	1709 (41.7)	2 (20.0)	
DLD (%)	0	218601 (52.2)	116 (47.3)	1512 (36.9)	7 (70.0)	<0.001
	1	200038 (47.8)	129 (52.7)	2587 (63.1)	3 (30.0)	
Malignancy (%)	0	348953 (83.4)	215 (87.8)	2790 (68.1)	10 (100.0)	<0.001
	1	69686 (16.6)	30 (12.2)	1309 (31.9)	0 (0.0)	
Varices (%)	0	387790 (92.6)	231 (94.3)	3488 (85.1)	10 (100.0)	<0.001
	1	30849 (7.4)	14 (5.7)	611 (14.9)	0 (0.0)	
Variceal_hemorrhage (%)	0	418183 (99.9)	244 (99.6)	4093 (99.9)	10 (100.0)	0.470
	1	456 (0.1)	1 (0.4)	6 (0.1)	0 (0.0)	

Ascites (%)	0	397751 (95.0)	234 (95.5)	3569 (87.1)	8 (80.0)	<0.001
	1	20888 (5.0)	11 (4.5)	530 (12.9)	2 (20.0)	
Hepatic_encephalopathy (%)	0	405604 (96.9)	236 (96.3)	3858 (94.1)	10 (100.0)	<0.001
	1	13035 (3.1)	9 (3.7)	241 (5.9)	0 (0.0)	
Hepatorenal_syndrome (%)	0	418425 (99.9)	245 (100.0)	4098 (100.0)	10 (100.0)	0.873
	1	214 (0.1)	0 (0.0)	1 (0.0)	0 (0.0)	
Spontaneous_bacterial_peritonitis(%)	0	418363 (99.9)	245 (100.0)	4094 (99.9)	10 (100.0)	0.554
	1	276 (0.1)	0 (0.0)	5 (0.1)	0 (0.0)	
HBV_antivirus_IFN (%)	0	418592 (100.0)	245 (100.0)	4090 (99.8)	10 (100.0)	<0.001
	1	47 (0.0)	0 (0.0)	9 (0.2)	0 (0.0)	
HBV_antivirus_Oral (%)	0	222875 (53.2)	11 (4.5)	2932 (71.5)	3 (30.0)	<0.001
	1	195764 (46.8)	234 (95.5)	1167 (28.5)	7 (70.0)	
HIV_antivirus (%)	0	418639 (100.0)	7 (2.9)	4099 (100.0)	3 (30.0)	<0.001
	1	0 (0.0)	238 (97.1)	0 (0.0)	7 (70.0)	
HCV_antivirus_IFN (%)	0	418592 (100.0)	245 (100.0)	4090 (99.8)	10 (100.0)	<0.001
	1	47 (0.0)	0 (0.0)	9 (0.2)	0 (0.0)	
HCV_antivirus_Oral (%)	0	418639 (100.0)	245 (100.0)	3632 (88.6)	10 (100.0)	<0.001
	1	0 (0.0)	0 (0.0)	467 (11.4)	0 (0.0)	
CCI_wo_HIV (%)	1	162940 (38.9)	102 (41.6)	746 (18.2)	7 (70.0)	<0.001
	2	255699 (61.1)	143 (58.4)	3353 (81.8)	3 (30.0)	
precise_CCI (%)	1	162940 (38.9)	102 (41.6)	746 (18.2)	7 (70.0)	<0.001
	2	101906 (24.3)	66 (26.9)	702 (17.1)	1 (10.0)	
	3	42802 (10.2)	25 (10.2)	460 (11.2)	0 (0.0)	
	4	39412 (9.4)	21 (8.6)	530 (12.9)	0 (0.0)	
	5	26756 (6.4)	10 (4.1)	438 (10.7)	0 (0.0)	
	6	44823 (10.7)	21 (8.6)	1223 (29.8)	2 (20.0)	

Variable_2018	level	HBV mono	HBV/HIV	HBV/HCV	HBV/HCV/HIV	p
n		441730	253	4244	10	
Sex (%)	1	255278 (57.8)	231 (91.3)	2377 (56.0)	8 (80.0)	<0.001
	2	186452 (42.2)	22 (8.7)	1867 (44.0)	2 (20.0)	
Age (%)	40	67406 (15.3)	43 (17.0)	319 (7.5)	2 (20.0)	<0.001
	40-50	110888 (25.1)	76 (30.0)	669 (15.8)	3 (30.0)	
	50-60	138934 (31.5)	78 (30.8)	1300 (30.6)	4 (40.0)	
	60-70	89053 (20.2)	44 (17.4)	1159 (27.3)	0 (0.0)	
	70-100	35449 (8.0)	12 (4.7)	797 (18.8)	1 (10.0)	
HCC (%)	0	412987 (93.5)	244 (96.4)	3713 (87.5)	10 (100.0)	<0.001
	1	28743 (6.5)	9 (3.6)	531 (12.5)	0 (0.0)	
Liver transplantation (%)	0	436789 (98.9)	251 (99.2)	4150 (97.8)	10 (100.0)	<0.001
	1	4941 (1.1)	2 (0.8)	94 (2.2)	0 (0.0)	
HTN (%)	0	308021 (69.7)	188 (74.3)	2269 (53.5)	5 (50.0)	<0.001
	1	133709 (30.3)	65 (25.7)	1975 (46.5)	5 (50.0)	
DM (%)	0	342409 (77.5)	195 (77.1)	2334 (55.0)	5 (50.0)	<0.001
	1	99321 (22.5)	58 (22.9)	1910 (45.0)	5 (50.0)	
DLD (%)	0	214509 (48.6)	117 (46.2)	1309 (30.8)	3 (30.0)	<0.001
	1	227221 (51.4)	136 (53.8)	2935 (69.2)	7 (70.0)	
Malignancy (%)	0	368861 (83.5)	228 (90.1)	3019 (71.1)	8 (80.0)	<0.001
	1	72869 (16.5)	25 (9.9)	1225 (28.9)	2 (20.0)	
Varices (%)	0	408872 (92.6)	241 (95.3)	3674 (86.6)	9 (90.0)	<0.001
	1	32858 (7.4)	12 (4.7)	570 (13.4)	1 (10.0)	
Variceal_hemorrhage (%)	0	441308 (99.9)	253 (100.0)	4238 (99.9)	10 (100.0)	0.759
	1	422 (0.1)	0 (0.0)	6 (0.1)	0 (0.0)	

Ascites (%)	0	420283 (95.1)	241 (95.3)	3763 (88.7)	7 (70.0)	<0.001
	1	21447 (4.9)	12 (4.7)	481 (11.3)	3 (30.0)	
Hepatic_encephalopathy (%)	0	427221 (96.7)	243 (96.0)	3967 (93.5)	10 (100.0)	<0.001
	1	14509 (3.3)	10 (4.0)	277 (6.5)	0 (0.0)	
Hepatorenal_syndrome (%)	0	441557 (100.0)	253 (100.0)	4238 (99.9)	10 (100.0)	0.011
	1	173 (0.0)	0 (0.0)	6 (0.1)	0 (0.0)	
Spontaneous_bacterial_peritonitis(%)	0	441464 (99.9)	253 (100.0)	4240 (99.9)	10 (100.0)	0.810
	1	266 (0.1)	0 (0.0)	4 (0.1)	0 (0.0)	
HBV_antivirus_IFN (%)	0	441711 (100.0)	253 (100.0)	4240 (99.9)	10 (100.0)	<0.001
	1	19 (0.0)	0 (0.0)	4 (0.1)	0 (0.0)	
HBV_antivirus_Oral (%)	0	234957 (53.2)	11 (4.3)	3106 (73.2)	4 (40.0)	<0.001
	1	206773 (46.8)	242 (95.7)	1138 (26.8)	6 (60.0)	
HIV_antivirus (%)	0	441730 (100.0)	6 (2.4)	4244 (100.0)	4 (40.0)	<0.001
	1	0 (0.0)	247 (97.6)	0 (0.0)	6 (60.0)	
HCV_antivirus_IFN (%)	0	441711 (100.0)	253 (100.0)	4240 (99.9)	10 (100.0)	<0.001
	1	19 (0.0)	0 (0.0)	4 (0.1)	0 (0.0)	
HCV_antivirus_Oral (%)	0	441730 (100.0)	253 (100.0)	3913 (92.2)	10 (100.0)	<0.001
	1	0 (0.0)	0 (0.0)	331 (7.8)	0 (0.0)	
CCI_wo_HIV (%)	1	164724 (37.3)	101 (39.9)	647 (15.2)	2 (20.0)	<0.001
	2	277006 (62.7)	152 (60.1)	3597 (84.8)	8 (80.0)	
precise_CCI (%)	1	164724 (37.3)	101 (39.9)	647 (15.2)	2 (20.0)	<0.001
	2	110222 (25.0)	71 (28.1)	720 (17.0)	2 (20.0)	
	3	48271 (10.9)	33 (13.0)	513 (12.1)	0 (0.0)	
	4	41805 (9.5)	12 (4.7)	544 (12.8)	1 (10.0)	
	5	28645 (6.5)	16 (6.3)	467 (11.0)	1 (10.0)	
	6	48063 (10.9)	20 (7.9)	1353 (31.9)	4 (40.0)	

Variable_2019	level	HBV mono	HBV/HIV	HBV/HCV	HBV/HCV/HIV	p
n		461708	293	4503	7	
Sex (%)	1	265034 (57.4)	267 (91.1)	2500 (55.5)	6 (85.7)	<0.001
	2	196674 (42.6)	26 (8.9)	2003 (44.5)	1 (14.3)	
Age (%)	40	64242 (13.9)	55 (18.8)	285 (6.3)	1 (14.3)	<0.001
	40-50	112625 (24.4)	79 (27.0)	670 (14.9)	3 (42.9)	
	50-60	144563 (31.3)	98 (33.4)	1280 (28.4)	1 (14.3)	
	60-70	99616 (21.6)	46 (15.7)	1342 (29.8)	1 (14.3)	
	70-100	40662 (8.8)	15 (5.1)	926 (20.6)	1 (14.3)	
HCC (%)	0	432412 (93.7)	286 (97.6)	4057 (90.1)	7 (100.0)	<0.001
	1	29296 (6.3)	7 (2.4)	446 (9.9)	0 (0.0)	
Liver transplantation (%)	0	456591 (98.9)	291 (99.3)	4417 (98.1)	7 (100.0)	<0.001
	1	5117 (1.1)	2 (0.7)	86 (1.9)	0 (0.0)	
HTN (%)	0	316349 (68.5)	218 (74.4)	2347 (52.1)	4 (57.1)	<0.001
	1	145359 (31.5)	75 (25.6)	2156 (47.9)	3 (42.9)	
DM (%)	0	353174 (76.5)	224 (76.5)	2069 (45.9)	5 (71.4)	<0.001
	1	108534 (23.5)	69 (23.5)	2434 (54.1)	2 (28.6)	
DLD (%)	0	215203 (46.6)	137 (46.8)	1230 (27.3)	2 (28.6)	<0.001
	1	246505 (53.4)	156 (53.2)	3273 (72.7)	5 (71.4)	
Malignancy (%)	0	384410 (83.3)	266 (90.8)	3067 (68.1)	5 (71.4)	<0.001
	1	77298 (16.7)	27 (9.2)	1436 (31.9)	2 (28.6)	
Varices (%)	0	427297 (92.5)	276 (94.2)	3968 (88.1)	7 (100.0)	<0.001
	1	34411 (7.5)	17 (5.8)	535 (11.9)	0 (0.0)	
Variceal_hemorrhage (%)	0	461251 (99.9)	292 (99.7)	4499 (99.9)	7 (100.0)	0.617
	1	457 (0.1)	1 (0.3)	4 (0.1)	0 (0.0)	

Ascites (%)	0	439776 (95.2)	276 (94.2)	4055 (90.1)	6 (85.7)	<0.001
	1	21932 (4.8)	17 (5.8)	448 (9.9)	1 (14.3)	
Hepatic_encephalopathy (%)	0	446197 (96.6)	285 (97.3)	4226 (93.8)	7 (100.0)	<0.001
	1	15511 (3.4)	8 (2.7)	277 (6.2)	0 (0.0)	
Hepatorenal_syndrome (%)	0	461536 (100.0)	293 (100.0)	4498 (99.9)	7 (100.0)	0.089
	1	172 (0.0)	0 (0.0)	5 (0.1)	0 (0.0)	
Spontaneous_bacterial_peritonitis(%)	0	461476 (99.9)	293 (100.0)	4499 (99.9)	7 (100.0)	0.690
	1	232 (0.1)	0 (0.0)	4 (0.1)	0 (0.0)	
HBV_antivirus_IFN (%)	0	461699 (100.0)	293 (100.0)	4503 (100.0)	7 (100.0)	0.993
	1	9 (0.0)	0 (0.0)	0 (0.0)	0 (0.0)	
HBV_antivirus_Oral (%)	0	243346 (52.7)	12 (4.1)	3465 (76.9)	2 (28.6)	<0.001
	1	218362 (47.3)	281 (95.9)	1038 (23.1)	5 (71.4)	
HIV_antivirus (%)	0	461708 (100.0)	11 (3.8)	4503 (100.0)	1 (14.3)	<0.001
	1	0 (0.0)	282 (96.2)	0 (0.0)	6 (85.7)	
HCV_antivirus_IFN (%)	0	461699 (100.0)	293 (100.0)	4503 (100.0)	7 (100.0)	0.993
	1	9 (0.0)	0 (0.0)	0 (0.0)	0 (0.0)	
HCV_antivirus_Oral (%)	0	461708 (100.0)	293 (100.0)	4164 (92.5)	7 (100.0)	<0.001
	1	0 (0.0)	0 (0.0)	339 (7.5)	0 (0.0)	
CCI_wo_HIV (%)	1	170618 (37.0)	116 (39.6)	638 (14.2)	1 (14.3)	<0.001
	2	291090 (63.0)	177 (60.4)	3865 (85.8)	6 (85.7)	
precise_CCI (%)	1	170618 (37.0)	116 (39.6)	638 (14.2)	1 (14.3)	<0.001
	2	114375 (24.8)	85 (29.0)	699 (15.5)	1 (14.3)	
	3	50706 (11.0)	28 (9.6)	510 (11.3)	1 (14.3)	
	4	44698 (9.7)	24 (8.2)	522 (11.6)	1 (14.3)	
	5	30260 (6.6)	12 (4.1)	531 (11.8)	1 (14.3)	
	6	51051 (11.1)	28 (9.6)	1603 (35.6)	2 (28.6)	

Variable_2020	level	HBV mono	HBV/HIV	HBV/HCV	HBV/HCV/HIV	p
n		457726	308	4062	2	
Sex (%)	1	262736 (57.4)	281 (91.2)	2236 (55.0)	1 (50.0)	<0.001
	2	194990 (42.6)	27 (8.8)	1826 (45.0)	1 (50.0)	
Age (%)	40	55601 (12.1)	48 (15.6)	267 (6.6)	0 (0.0)	<0.001
	40-50	109708 (24.0)	83 (26.9)	561 (13.8)	0 (0.0)	
	50-60	141528 (30.9)	107 (34.7)	1133 (27.9)	1 (50.0)	
	60-70	107058 (23.4)	46 (14.9)	1219 (30.0)	0 (0.0)	
	70-100	43831 (9.6)	24 (7.8)	882 (21.7)	1 (50.0)	
HCC (%)	0	428335 (93.6)	299 (97.1)	3640 (89.6)	2 (100.0)	<0.001
	1	29391 (6.4)	9 (2.9)	422 (10.4)	0 (0.0)	
Liver transplantation (%)	0	452278 (98.8)	306 (99.4)	3973 (97.8)	2 (100.0)	<0.001
	1	5448 (1.2)	2 (0.6)	89 (2.2)	0 (0.0)	
HTN (%)	0	306631 (67.0)	213 (69.2)	2042 (50.3)	1 (50.0)	<0.001
	1	151095 (33.0)	95 (30.8)	2020 (49.7)	1 (50.0)	
DM (%)	0	345240 (75.4)	234 (76.0)	1803 (44.4)	1 (50.0)	<0.001
	1	112486 (24.6)	74 (24.0)	2259 (55.6)	1 (50.0)	
DLD (%)	0	205348 (44.9)	118 (38.3)	1032 (25.4)	1 (50.0)	<0.001
	1	252378 (55.1)	190 (61.7)	3030 (74.6)	1 (50.0)	
Malignancy (%)	0	380344 (83.1)	273 (88.6)	2705 (66.6)	0 (0.0)	<0.001
	1	77382 (16.9)	35 (11.4)	1357 (33.4)	2 (100.0)	
Varices (%)	0	423263 (92.5)	283 (91.9)	3557 (87.6)	2 (100.0)	<0.001
	1	34463 (7.5)	25 (8.1)	505 (12.4)	0 (0.0)	
Variceal_hemorrhage (%)	0	457296 (99.9)	306 (99.4)	4055 (99.8)	2 (100.0)	0.006
	1	430 (0.1)	2 (0.6)	7 (0.2)	0 (0.0)	

Ascites (%)	0	436065 (95.3)	297 (96.4)	3611 (88.9)	1 (50.0)	<0.001
	1	21661 (4.7)	11 (3.6)	451 (11.1)	1 (50.0)	
Hepatic_encephalopathy (%)	0	443248 (96.8)	296 (96.1)	3841 (94.6)	2 (100.0)	<0.001
	1	14478 (3.2)	12 (3.9)	221 (5.4)	0 (0.0)	
Hepatorenal_syndrome (%)	0	457542 (100.0)	308 (100.0)	4060 (100.0)	2 (100.0)	0.977
	1	184 (0.0)	0 (0.0)	2 (0.0)	0 (0.0)	
Spontaneous_bacterial_peritonitis(%)	0	457521 (100.0)	308 (100.0)	4055 (99.8)	2 (100.0)	0.002
	1	205 (0.0)	0 (0.0)	7 (0.2)	0 (0.0)	
HBV_antivirus_IFN (%)	0	457724 (100.0)	308 (100.0)	4062 (100.0)	2 (100.0)	0.999
	1	2 (0.0)	0 (0.0)	0 (0.0)	0 (0.0)	
HBV_antivirus_Oral (%)	0	231552 (50.6)	17 (5.5)	3068 (75.5)	1 (50.0)	<0.001
	1	226174 (49.4)	291 (94.5)	994 (24.5)	1 (50.0)	
HIV_antivirus (%)	0	457726 (100.0)	15 (4.9)	4062 (100.0)	1 (50.0)	<0.001
	1	0 (0.0)	293 (95.1)	0 (0.0)	1 (50.0)	
HCV_antivirus_IFN (%)	0	457724 (100.0)	308 (100.0)	4062 (100.0)	2 (100.0)	0.999
	1	2 (0.0)	0 (0.0)	0 (0.0)	0 (0.0)	
HCV_antivirus_Oral (%)	0	457726 (100.0)	308 (100.0)	3849 (94.8)	2 (100.0)	<0.001
	1	0 (0.0)	0 (0.0)	213 (5.2)	0 (0.0)	
CCI_wo_HIV (%)	1	185389 (40.5)	133 (43.2)	611 (15.0)	0 (0.0)	<0.001
	2	272337 (59.5)	175 (56.8)	3451 (85.0)	2 (100.0)	
precise_CCI (%)	1	185389 (40.5)	133 (43.2)	611 (15.0)	0 (0.0)	<0.001
	2	106006 (23.2)	72 (23.4)	600 (14.8)	0 (0.0)	
	3	43834 (9.6)	29 (9.4)	427 (10.5)	0 (0.0)	
	4	44936 (9.8)	30 (9.7)	501 (12.3)	0 (0.0)	
	5	29014 (6.3)	11 (3.6)	477 (11.7)	0 (0.0)	
	6	48547 (10.6)	33 (10.7)	1446 (35.6)	2 (100.0)	

Variable_2021	level	HBV mono	HBV/HIV	HBV/HCV	HBV/HCV/HIV	p
n		469834	297	3672	8	
Sex (%)	1	267688 (57.0)	269 (90.6)	2018 (55.0)	6 (75.0)	<0.001
	2	202146 (43.0)	28 (9.4)	1654 (45.0)	2 (25.0)	
Age (%)	40	50999 (10.9)	48 (16.2)	248 (6.8)	1 (12.5)	<0.001
	40-50	111242 (23.7)	79 (26.6)	450 (12.3)	4 (50.0)	
	50-60	142101 (30.2)	90 (30.3)	986 (26.9)	1 (12.5)	
	60-70	117735 (25.1)	58 (19.5)	1162 (31.6)	1 (12.5)	
	70-100	47757 (10.2)	22 (7.4)	826 (22.5)	1 (12.5)	
HCC (%)	0	440424 (93.7)	282 (94.9)	3284 (89.4)	7 (87.5)	<0.001
	1	29410 (6.3)	15 (5.1)	388 (10.6)	1 (12.5)	
Liver transplantation (%)	0	464176 (98.8)	293 (98.7)	3595 (97.9)	8 (100.0)	<0.001
	1	5658 (1.2)	4 (1.3)	77 (2.1)	0 (0.0)	
HTN (%)	0	309513 (65.9)	204 (68.7)	1825 (49.7)	6 (75.0)	<0.001
	1	160321 (34.1)	93 (31.3)	1847 (50.3)	2 (25.0)	
DM (%)	0	347989 (74.1)	225 (75.8)	1529 (41.6)	6 (75.0)	<0.001
	1	121845 (25.9)	72 (24.2)	2143 (58.4)	2 (25.0)	
DLD (%)	0	199251 (42.4)	107 (36.0)	900 (24.5)	5 (62.5)	<0.001
	1	270583 (57.6)	190 (64.0)	2772 (75.5)	3 (37.5)	
Malignancy (%)	0	388779 (82.7)	260 (87.5)	2449 (66.7)	5 (62.5)	<0.001
	1	81055 (17.3)	37 (12.5)	1223 (33.3)	3 (37.5)	
Varices (%)	0	433845 (92.3)	272 (91.6)	3211 (87.4)	8 (100.0)	<0.001
	1	35989 (7.7)	25 (8.4)	461 (12.6)	0 (0.0)	
Variceal_hemorrhage (%)	0	469402 (99.9)	297 (100.0)	3664 (99.8)	8 (100.0)	0.089
	1	432 (0.1)	0 (0.0)	8 (0.2)	0 (0.0)	

Ascites (%)	0	448086 (95.4)	281 (94.6)	3318 (90.4)	7 (87.5)	<0.001
	1	21748 (4.6)	16 (5.4)	354 (9.6)	1 (12.5)	
Hepatic_encephalopathy (%)	0	453873 (96.6)	285 (96.0)	3453 (94.0)	7 (87.5)	<0.001
	1	15961 (3.4)	12 (4.0)	219 (6.0)	1 (12.5)	
Hepatorenal_syndrome (%)	0	469664 (100.0)	297 (100.0)	3670 (99.9)	8 (100.0)	0.930
	1	170 (0.0)	0 (0.0)	2 (0.1)	0 (0.0)	
Spontaneous_bacterial_peritonitis(%)	0	469661 (100.0)	297 (100.0)	3669 (99.9)	8 (100.0)	0.554
	1	173 (0.0)	0 (0.0)	3 (0.1)	0 (0.0)	
HBV_antivirus_IFN (%)	0	469834 (100.0)	297 (100.0)	3672 (100.0)	8 (100.0)	NA
	1	0 (0.0)	0 (0.0)	0 (0.0)	0 (0.0)	
HBV_antivirus_Oral (%)	0	235149 (50.0)	10 (3.4)	2727 (74.3)	1 (12.5)	<0.001
	1	234685 (50.0)	287 (96.6)	945 (25.7)	7 (87.5)	
HIV_antivirus (%)	0	469834 (100.0)	4 (1.3)	3672 (100.0)	1 (12.5)	<0.001
	1	0 (0.0)	293 (98.7)	0 (0.0)	7 (87.5)	
HCV_antivirus_IFN (%)	0	469834 (100.0)	297 (100.0)	3672 (100.0)	8 (100.0)	NA
	1	0 (0.0)	0 (0.0)	0 (0.0)	0 (0.0)	
HCV_antivirus_Oral (%)	0	469834 (100.0)	297 (100.0)	3508 (95.5)	8 (100.0)	<0.001
	1	0 (0.0)	0 (0.0)	164 (4.5)	0 (0.0)	
CCI_wo_HIV (%)	1	191964 (40.9)	139 (46.8)	559 (15.2)	3 (37.5)	<0.001
	2	277870 (59.1)	158 (53.2)	3113 (84.8)	5 (62.5)	
precise_CCI (%)	1	191964 (40.9)	139 (46.8)	559 (15.2)	3 (37.5)	<0.001
	2	107423 (22.9)	66 (22.2)	506 (13.8)	0 (0.0)	
	3	44205 (9.4)	24 (8.1)	380 (10.3)	0 (0.0)	
	4	47193 (10.0)	19 (6.4)	457 (12.4)	3 (37.5)	
	5	29957 (6.4)	17 (5.7)	467 (12.7)	1 (12.5)	
	6	49092 (10.4)	32 (10.8)	1303 (35.5)	1 (12.5)	

HTN, hypertension; DM, diabetes; DLD, dyslipidemia; IFN, interferon; NAs, nucleos(t)ide analogue; ART, antiretroviral therapy; DAA, direct acting antiviral agent; CCI, charlson comorbidity index; Deyo–-Charlson Comorbidity Index was calculated excluding HIV disease score; p-values were calculated from one-way ANOVA with repeated measures for continuous variables and generalized estimating equations for categorical variables between HBV monoinfection and matched controls
